# The pectoralis major index: a novel computed tomography biomarker for low thoracic muscle mass and eastern cooperative oncology group performance status in lung cancer patients

**DOI:** 10.3389/fnut.2026.1712633

**Published:** 2026-02-24

**Authors:** Wenbin Gu, Yusheng Li, Fang Wang, Chunyu Wu, Zhaojie Peng, Xihua Zhou, Wenxuan Lei, Mingxuan Huang, Fei Peng

**Affiliations:** Department of Radiology, The First Affiliated Hospital, Hengyang Medical School, University of South China, Hengyang, China

**Keywords:** computed tomography, fourth thoracic vertebra, low thoracic muscle mass, lung cancer, pectoralis major index

## Abstract

**Background:**

Chest computed tomography (CT) seldom covers the third lumbar (L3) vertebral level, the standard landmark for assessing total body muscle mass. As muscle measurements at the fourth thoracic (T4) level show high concordance with those at L3, the T4 level may serve as a viable alternative. We evaluated the discriminatory performance of T4 musculature for lung cancer–related low thoracic muscle mass (LTMM) and its association with Eastern Cooperative Oncology Group (ECOG) performance status.

**Methods:**

We retrospectively included 289 inpatients with newly diagnosed lung cancer who underwent chest CT within 3 months. At T4, the pectoralis major, pectoralis minor, and chest–wall muscle group were segmented to derive cross–sectional area (CSA), density, and height–normalized indices (cm^2^/m^2^). Low thoracic muscle mass (LTMM) was defined by sex–specific 25th–percentile thoracic 4th vertebra level muscle index (T4MI) cutoffs (40.78 cm^2^/m^2^ for men, 33.15 cm^2^/m^2^ for women). Patients were stratified by sex and clinical stage (I–II early–stage vs. III–IV advanced–stage). ROC analyses compared discriminatory performance; logistic regression tested associations with poor ECOG performance status.

**Results:**

Compared with non–low thoracic muscle mass (non–LTMM), low thoracic muscle mass (LTMM) group showed smaller CSA, lower density, and reduced indices. Across muscle groups, indices outperformed CSA and density. The pectoralis major index achieved the highest AUC in the overall cohort (AUC = 0.833) and reached excellent discrimination in the overall male cohort (AUC = 0.922). Notably, stage–stratified analyses showed consistently superior discriminatory performance for the pectoralis major index in the male population (early–stage AUC = 0.917, advanced–stage AUC = 0.912). In addition, the pectoralis major index was independently associated with poor ECOG performance status (odds ratio = 0.932, *p* = 0.009).

**Conclusion:**

T4-level muscle metrics showed good internal discrimination for lung cancer–related low thoracic muscle mass across sex and disease stage, with the pectoralis major index achieving the highest overall performance. Moreover, the pectoralis major index may be a potential imaging biomarker associated with ECOG performance status.

## 1 Introduction

Lung cancer is one of the most common malignancies worldwide and a leading cause of cancer mortality (1). Tumor–driven systemic inflammation, dysregulated energy metabolism, and accelerated proteolysis promote skeletal–muscle loss, culminating in low muscle mass and related complications (2, 3). In lung cancer cohorts, the reported prevalence of sarcopenia ranges from 35 to 74%, and its presence consistently predicts poorer outcomes (4).

The third lumbar vertebral level (L3) serves as the conventional diagnostic landmark in international sarcopenia research (5). Nevertheless, in routine clinical practice and health screening, individuals undergoing check-ups and patients with lung cancer or other cardiothoracic diseases often receive chest CT alone; thus, the L3 level is frequently outside the scan range, precluding single-examination assessment of whole-body muscle mass based on L3-level musculature (6). This practical constraint has shifted attention toward thoracic muscle metrics available on routine chest CT. Studies have shown that reductions in thoracic muscle mass have been linked to disease progression, and mortality in chronic obstructive pulmonary disease (COPD), idiopathic pulmonary fibrosis (IPF), lung transplantation, COVID–19 and lung cancer (7–11). Particularly, the T4 skeletal muscle area and index show strong concordance with their L3 counterparts and thus have viable substitutive value (12–14). Anatomically, owing to its relatively large size, consistent anatomy, and well–defined fascial boundaries, the pectoralis major is well suited for reproducible quantification on routine axial chest CT at the T4 level. Mechanistically, cancer–related inflammation and catabolic metabolism can reduce thoracic muscle quality, limiting physiologic reserve and thereby plausibly contributing to poorer Eastern Cooperative Oncology Group (ECOG) performance status. Pectoralis–derived CT metrics have shown biologic relevance to lean mass and have been associated with prognosis in non-small cell lung cancer (NSCLC), supporting it as a valuable prognostic indicator (11, 15). We therefore hypothesize that the pectoralis major can serve as a representative T4 muscle to help distinguish low from non–low thoracic muscle mass (non–LTMM) status in lung cancer and to provide additional imaging information related to performance status. Subsequently, this may facilitate a “one–stop–shop” integrated evaluation of tumor burden and functional status within a single thoracic examination.

Multiple factors, including clinical tumor stage (16), treatment modality (17), and sarcopenia (4), have been recognized as risk factors for poor prognosis in patients with lung cancer, with skeletal muscle mass being particularly prominent. Importantly, the pectoralis muscle index, derived from muscle mass, demonstrates predictive utility for clinical outcomes. For example, in localized NSCLC after curative–intent surgery, lower pectoralis muscle index was associated with worse 5–year overall survival (64.2% vs. 86.7%) and remained independently associated with mortality, with a 2.09–fold higher hazard of death (11). Given the likely pivotal role of the pectoralis major within thoracic musculature, we speculate that the pectoralis major index has potential value for discriminating low thoracic muscle mass (LTMM) and predicting performance status in patients with lung cancer.

Our objective was to analyze T4–level muscle parameters in patients with lung cancer to evaluate the discriminatory performance of these metrics for distinguishing low versus non–low thoracic muscle mass status across sex and disease-stage strata (early-stage vs. advanced-stage). We also aimed to examine associations between thoracic musculature parameters and patients’ ECOG performance status.

## 2 Materials and methods

### 2.1 Study design and participants

**Figure 1:**
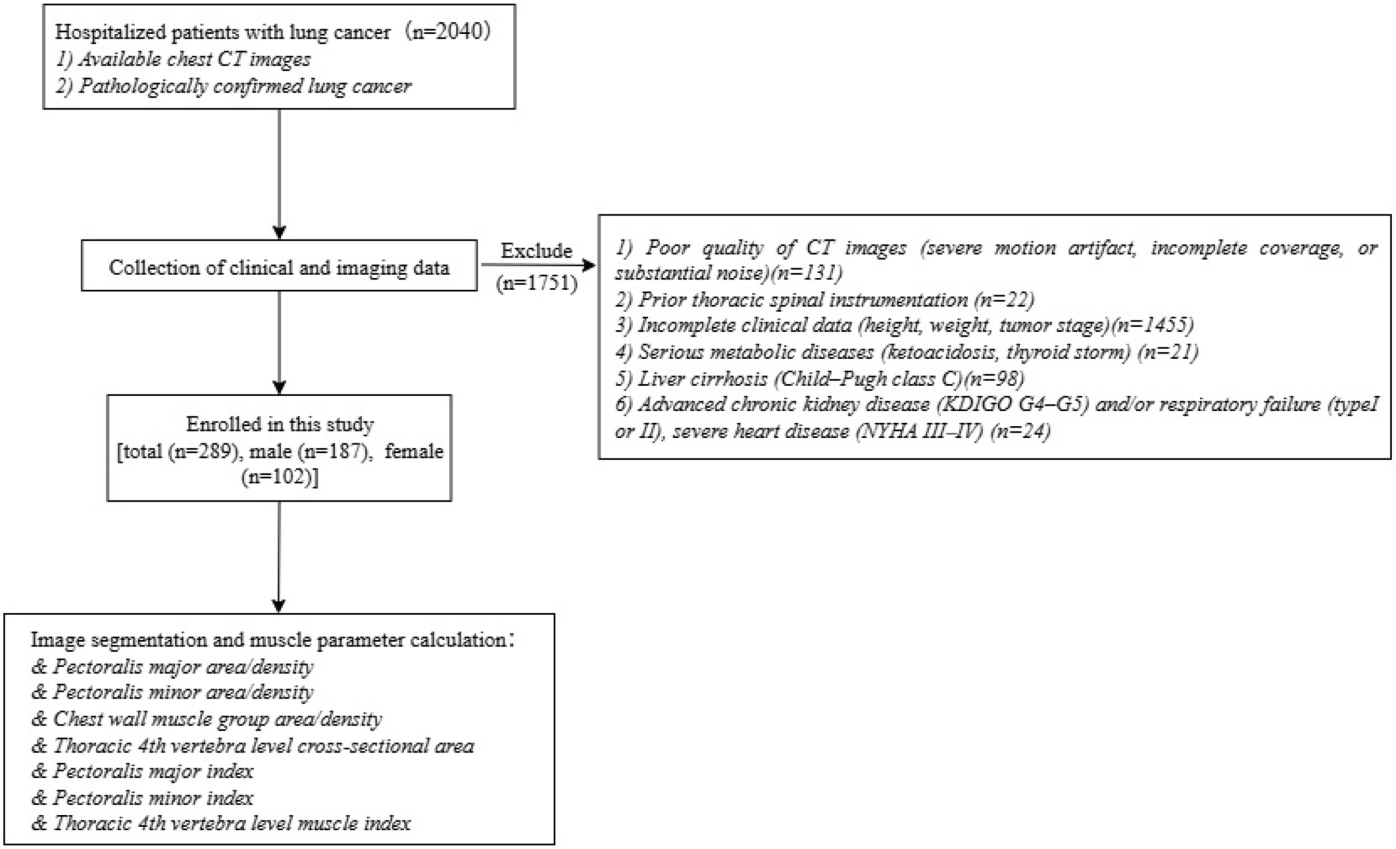
Flowchart for inclusion and exclusion of patients with lung cancer in this study. CT, computed tomography; KDIGO, Kidney Disease: Improving Global Outcomes; NYHA, New York Heart Association.

This retrospective study was approved by the Research Ethics Committee of the First Affiliated Hospital of the University of South China. From January 2018 to December 2023, all inpatients at our institution with newly diagnosed lung cancer were assessed for eligibility (*n* = 2,040). Patients were included if they: (1) had lung cancer confirmed for the first time by pathology or cytology; and (2) underwent chest CT at our institution within 3 months of admission, with complete image coverage and adequate quality for quantitative analysis of thoracic muscle parameters. Exclusion criteria were: (1) CT images of insufficient quality for accurate measurement (severe motion artifact, incomplete coverage, or substantial noise) (*n* = 131); (2) prior thoracic spinal instrumentation that could distort T4–level anatomy and compromise thoracic muscle assessment (*n* = 22); (3) missing key clinical variables required for analysis (height, weight, tumor stage) (*n* = 1,455); (4) severe metabolic disorders (diabetic ketoacidosis or thyroid storm) (*n* = 21); (5) advanced liver cirrhosis (Child–Pugh class C) (*n* = 98); and (6) conditions likely to markedly alter systemic muscle status independent of lung cancer, including advanced chronic kidney disease (KDIGO G4–G5) and/or respiratory failure (type I or II), as well as severe cardiac disease (New York Heart Association class III–IV) (*n* = 24). After excluding 1,751 patients, a total of 289 patients were ultimately included (Figure 1).

### 2.2 CT examinations

Chest CT was performed in the head–first supine position with both arms elevated overhead, during an end–inspiratory breath–hold. Scans were obtained on Siemens SOMATOM Definition AS/Force/Perspective/Definition Flash systems (Siemens Healthineers, Erlangen, Germany). Parameters: 120 kVp, automatic tube–current modulation; reference range 100–300 mA; 5–mm sections with 1–mm reconstructed images. To minimize potential inter–scanner variability, all examinations were performed under a harmonized institutional chest CT protocol. For quantitative analyses, we exclusively used the 1–mm axial reconstruction series with standard soft–tissue settings, and the scanner model was recorded for each scan as part of quality control.

### 2.3 CT image analysis

**Figure 2:**
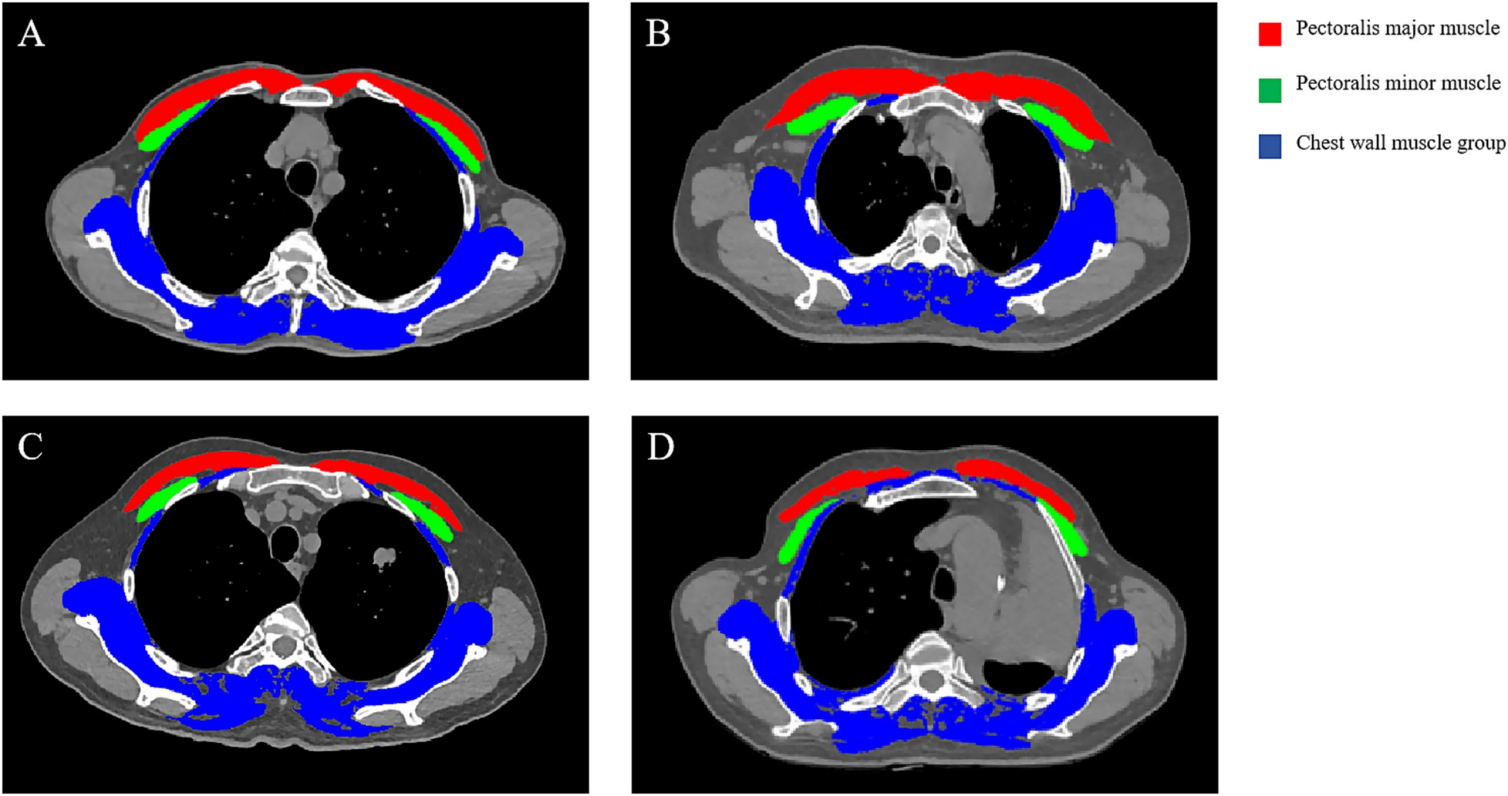
Representative T4–level chest CT images across clinical stages of lung cancer. **(A)** Stage I, **(B)** Stage II, **(C)** Stage III, and **(D)** Stage IV. Colored overlays indicate segmented muscles: red, pectoralis major; green, pectoralis minor; blue, chest wall muscle group (e.g., serratus anterior, intercostals, teres major, subscapularis, erector spinae, trapezius). T4, thoracic vertebra 4. CT, computed tomography.

Muscle delineation and quantitative measurements were conducted exclusively on unenhanced chest CT images. The measurement level was defined as the axial slice at the fourth thoracic vertebra (T4). On CT, the bilateral pectoralis major, pectoralis minor, and the chest wall muscle group (including the serratus anterior, external intercostals, teres major, subscapularis, erector spinae, and trapezius) were identified and measured using the sliceOmatic version 5.0 Rev–9 (TomoVision, Magog, Canada) image analysis software based on the established Hounsfield unit (HU) threshold for muscle tissue (−29 to +150HU) (5) (Figure 2). Segmentation was performed by a single radiologist with 3 years of experience (XX) under the supervision of a musculoskeletal radiologist with 10 years of experience (XX) as part of quality control. The operator was blinded to clinical outcomes and group assignment to minimize bias. The total muscle area at T4 (pectorals plus chest wall group) was normalized to height^2^ to yield the thoracic 4th vertebra level muscle index (T4MI, expressed as cm^2^/m^2^). Similarly, the cross–sectional areas of the pectoralis major and pectoralis minor were normalized to height^2^ to derive the pectoralis major index and pectoralis minor index, respectively. Finally, the average value calculated using the outlined ROIs on three consecutive slices was used to determine each muscle parameter. LTMM was defined using sex–specific 25th–percentile T4MI cutoffs as follows: 40.78 cm^2^/m^2^ for men and 33.15 cm^2^/m^2^ for women. This lowest–quartile approach is similar to that used in prior thoracic CT body–composition studies, and our cutoffs fall within the range reported in published cohorts (7, 14, 18). In this study, these percentile–based cutoffs were applied for exploratory, within–cohort stratification rather than as externally validated diagnostic thresholds.

### 2.4 Reproducibility

Intra-observer reproducibility was performed for CT measurements. The ICCs ranges for muscle area, muscle density, and muscle index were 0.981–0.994 (95% CIs: 0.977–0.996), 0.967–0.990 (95% CIs: 0.959–0.992), 0.971–0.991 (95% CIs: 0.959–0.994) respectively, indicating excellent reproducibility.

### 2.5 TNM staging of lung cancer

Tumor stage was determined from pathologic findings and classified as stages I–IV according to the AJCC 8th–edition TNM (Tumor Node Metastasis) system (17). Patients with stage I–II disease considered eligible for surgical resection were categorized as the early–stage group, whereas those with stage III–IV disease were categorized as the advanced–stage group (19).

### 2.6 Eastern cooperative oncology group (ECOG) scores

All patients underwent performance status assessment at admission using the Eastern Cooperative Oncology Group (ECOG) scale (20). ECOG scores range from 0 to 5, with higher scores indicating poorer function. 0 score, fully active without restrictions; 1 score, restricted in strenuous activity but ambulatory and able to perform light work; 2 score, ambulatory and capable of all self–care but unable to work, up and about for >50% of waking hours; 3 score, limited self–care and confined to bed or chair for >50% of waking hours; 4 score, completely disabled, unable to perform self–care, and totally confined to bed or chair; 5 score, death. An ECOG score of 0–1 is defined as good performance status, whereas an ECOG score of ≥2 is defined as poor performance status (21).

### 2.7 Statistical analysis

Normality was assessed with the Shapiro–Wilk test. Continuous variables are summarized as median (interquartile range, IQR) and categorical variables as count (percentage). Between–group comparisons used the Mann–Whitney U test for continuous variables and Pearson *χ*^2^ or Fisher exact tests for categorical variables. Effect sizes are reported as rank–biserial correlation for continuous variables and Cramér’s V for categorical variables. The cohort was stratified by sex and by clinical stage (early vs. advanced). Within each subgroup, receiver operating characteristic (ROC) curves were generated to evaluate the discriminatory performance of the T4 muscle parameters for distinguishing low versus non–low thoracic muscle mass status. Sex–stratified analyses were performed descriptively, and no formal sex–by–parameter interaction testing was conducted. Therefore, any between–sex differences should be interpreted as exploratory. Using ECOG score as the dependent variable, clinical characteristics together with T4 muscle group parameters were entered as independent variables in logistic regression to estimate odds ratios (ORs). Given the small number of patients with ECOG 3 and the potential instability of multivariable ordinal regression, we modeled ECOG as a binary outcome (0–1 vs. ≥2) and performed multivariable binary logistic regression analysis. Prior to multivariable modeling, multicollinearity among candidate predictors was assessed using variance inflation factors (VIFs). Variables significant in univariable analyses were subsequently included in multivariable models to assess independent associations. Model calibration was evaluated using the Hosmer–Lemeshow goodness–of–fit test and the Brier score. Analyses were conducted with SPSS version 27.0 (IBM Corp., Armonk, NY, USA), GraphPad Prism version 10.0 (GraphPad Software, San Diego, CA, USA), and MedCalc Statistical Software version 15.2.2 (MedCalc Software Ltd., Ostend, Belgium). Two–sided *p* values < 0.05 were considered statistically significant.

## 3 Results

### 3.1 Participant characteristics

Table 1 presents demographic characteristics for the final analytic participants. A total of 289 patients with lung cancer were stratified into low thoracic muscle mass (LTMM) and non–low thoracic muscle mass (non–LTMM) groups using sex–specific 25th–percentile T4MI cutoffs. Of these, 218 patients (75.4%) were with non–LTMM and 71 patients (24.6%) were with LTMM. Between–group differences were significant for age, weight, and BMI (all *p* < 0.05). No significant differences were observed in sex distribution, height, smoking history, diabetes mellitus, cardiovascular history, or ECOG performance status (all *p* > 0.05). Although ECOG performance status did not differ significantly between LTMM and non–LTMM groups in unadjusted comparisons, associations between ECOG and thoracic muscle parameters were observed in our multivariable logistic regression models.

**Table 1:** Characteristics of the study participants.

Variable	Non–LTMM (*n* = 218)	LTMM (*n* = 71)	*p*	Effect sizes
Age (median, IQR)	60 (55–67)	67 (59–72)	<0.001	0.356
Sex (male/female)	141/77	46/25	0.987	0.001
Height (cm, IQR)	164.00 (157.75–168.25)	165.00 (159.00–170.00)	0.262	0.088
Weight (kg, IQR)	56.75 (51.68–64.20)	54.50 (47.00–60.00)	0.005	−0.222
BMI (kg/m^2^)	21.50 (19.60–23.70)	19.72 (18.49–21.71)	<0.01	−0.349
Smoking status (*n*, %)	82 (38%)	25 (35%)	0.716	0.021
Diabetes (*n*, %)	7 (3%)	2 (2%)	0.868	0.01
Coronary heart disease (*n*, %)	7 (3%)	3 (4%)	0.685	0.024
Adenocarcinoma (*n*, %)	158 (72%)	52 (73%)	0.899	0.052
ECOG (0/1/2/3), *n*	33/150/31/4	8/45/16/2	0.089	0.103

Effect sizes are rank–biserial correlation (r_rb) for continuous variables and Cramér’s V for categorical variables. IQR, interquartile range; BMI, body mass index; ECOG PS, Eastern Cooperative Oncology Group performance status; Non–LTMM, non–low thoracic muscle mass; LTMM, low thoracic muscle mass; sex is displayed as “male/female,” n/n.

### 3.2 Measurements of muscle parameters

Table 2 summarizes subgroup comparisons of the pectoralis major, pectoralis minor, and chest wall muscle group by cross–sectional area, density, and skeletal muscle index. Because sex–specific cutoffs were used to define LTMM, patients were first stratified into male and female non–LTMM and LTMM groups: male non–LTMM (*n* = 141) and LTMM (*n* = 46); female non–LTMM (*n* = 77) and LTMM (*n* = 25). Across the overall cohort, both in males and females, the LTMM group showed significantly lower pectoralis major/minor and chest wall muscle area and density, as well as lower pectoralis major and minor indices, compared with the non–LTMM group (all *p* < 0.05).

**Table 2:** Thoracic musculature at T4 by sex and clinical stage.

Parameters	Male stage I–IV (*n* = 187)		Female stage I–IV (*n* = 102)		Male (*n* = 187)				Female (*n* = 102)				All subjects (*n* = 289)	
					Stage I–II (*n* = 53)		Stage III–IV (*n* = 134)		Stage I–II (*n* = 45)		Stage III–IV (*n* = 57)		Non–LTMM (*n* = 218)	LTMM (*n* = 71)
	Non–LTMM (*n* = 141)	LTMM (*n* = 46)	Non–LTMM (*n* = 77)	LTMM (*n* = 25)	Non–LTMM (*n* = 48)	LTMM (*n* = 5)	Non–LTMM (*n* = 93)	LTMM (*n* = 41)	Non–LTMM (*n* = 35)	LTMM (*n* = 10)	Non–LTMM (*n* = 42)	LTMM (*n* = 15)		
Pectoralis major area (cm^2^)	28.99 (26.09–33.25)	21.24(18.56–25.21)^a^	18.24(15.88–21.42)	13.84(10.69–15.19)^b^	29.76(26.02–34.43)	19.80(15.60–26.89)^c^	28.88(26.07–32.88)	21.75(18.65–25.29)^d^	18.16(15.87–21.49)	13.00(9.55–14.68)^e^	18.34(16.17–21.05)	14.03(12.19–15.66)^f^	26.03(20.41–30.81)	18.56(14.34–23.22)^g^
Pectoralis minor area (cm^2^)	9.53(8.45–10.77)	7.74(6.44–8.55)^a^	6.89(6.09–7.77)	5.53(4.39–6.06)^b^	9.22(8.61–10.28)	7.69(5.97–8.34)^c^	9.80(8.29–10.80)	7.79(6.48–8.85)^d^	6.91(6.16–7.59)	5.75(4.91–6.19)^e^	6.84(6.06–7.94)	5.49(4.25–5.89)^f^	8.64(7.27–10.03)	6.52(5.78–8.35)^g^
Chest wall muscle group area (cm^2^)	91.06(86.55–99.89)	76.47(71.15–81.41)^a^	68.35(60.74–74.51)	54.41(49.68–57.02)^b^	92.78(87.48–106.20)	81.71(65.86–82.55)^c^	90.66(85.56–98.81)	76.14(70.98–80.92)^d^	68.15(62.59–74.19)	56.35(54.04–59.16)^e^	68.57(59.87–75.14)	53.22(46.29–56.61)^f^	86.55(71.56–95.63)	70.64(56.51–79.03)^g^
Pectoralis major density (HU)	44.48(40.09–49.43)	39.33(35.00–44.41)^a^	38.21(29.60–42.95)	35.16(22.50–37.11)^b^	45.32(40.63–50.44)	42.97(33.25–48.14)	44.14(38.83–48.75)	39.06(34.95–44.32)^d^	38.21(31.29–42.79)	28.30(20.70–35.65)^e^	38.28(27.56–43.00)	36.01(33.30–39.74)	42.66(36.58–48.02)	37.13(31.41–43.47)^g^
Pectoralis minor density (HU)	43.64(39.38–48.99)	41.34(37.07–45.36)^a^	39.95(36.84–44.13)	36.49(32.28–41.15)^b^	45.17(39.63–50.16)	42.49(37.88–47.68)	42.64(39.27–48.17)	41.03(36.84–45.36)^d^	42.69(36.99–44.95)	37.06(29.85–38.70)^e^	39.65(35.28–42.91)	36.31(32.37–43.44)	42.66(37.87–47.49)	39.45(35.15–43.44)^g^
Chest wall muscle group density (HU)	43.74(40.59–47.18)	40.02(37.90–43.31)^a^	40.96(37.57–44.44)	38.84(36.27–40.97)^b^	44.73(41.64–47.55)	43.75(38.23–45.66)	43.42(39.94–46.68)	39.56(37.80–42.73)^d^	41.88(38.01–45.07)	38.40(36.37–40.99)^e^	38.97(31.52–42.54)	38.84(33.53–40.96)	43.23(39.13–46.11)	39.28(36.81–42.36)^g^
Pectoralis major index (cm^2^/m^2^)	10.62(9.32–12.06)	7.64(6.77–8.54)^a^	7.47(6.60–8.85)	5.41(4.36–6.30)^b^	10.62(9.35–12.55)	6.85(6.31–9.18)^c^	10.63(9.25–11.81)	7.67(6.80–8.54)^d^	7.25(6.60–9.53)	5.36(4.03–5.83)^e^	7.53(6.54–8.70)	5.99(4.53–6.56)^f^	9.54(8.10–11.19)	6.84(5.82–8.02)^g^
Pectoralis minor index (cm^2^/m^2^)	3.40(3.04–3.95)	2.71(2.38–3.07)^a^	2.80(2.55–3.19)	2.15(1.85–2.47)^b^	3.33(3.03–3.92)	2.75(2.39–2.85)^c^	3.45(3.04–3.96)	2.63(2.38–3.07)^d^	2.80(2.54–3.12)	2.17(2.02–2.50)^e^	2.79(2.56–3.27)	2.12(1.80–2.50)^f^	3.20(2.82–3.72)	2.49(2.13–2.87)^g^
T4MI (cm^2^/m^2^)	47.06(44.11–51.27)	37.95(36.01–39.44)^a^	37.05(34.77–40.76)	30.44(27.48–31.66)^b^	48.25(44.17–53.38)	37.86(35.40–40.24)^c^	46.46(44.08–50.77)	37.99(36.19–39.33)^d^	37.65(35.54–40.78)	30.02(29.17–31.61)^e^	36.56(34.39–40.84)	30.67(26.22–32.17)^f^	44.30(40.34–49.06)	35.95(31.20–38.91)^g^
T4CSA (cm^2^)	130.88(121.66–141.67)	107.05(98.14–114.30)^a^	92.80(84.69–101.77)	73.13(66.50–77.88)^b^	130.74(124.36–151.06)	109.42(87.42–117.67)^c^	131.15(120.92–139.89)	106.97(98.50–113.49)^d^	92.42(85.73–103.05)	74.44(70.23–80.03)^e^	93.99(84.28–101.59)	73.13(61.52–75.33)^f^	121.14(99.03–135.67)	98.09 (76.20–109.89)^g^

^a^*p* < 0.05, male stage I–IV non–LTMM vs. LTMM; ^b^*p* < 0.05, female stage I–IV non–LTMM vs. LTMM; ^c^*p* < 0.05, male stage I–II non–LTMM vs. LTMM; ^d^*p* < 0.05, male stage III–IV non–LTMM vs. LTMM; ^e^*p* < 0.05, female stage I–II non–LTMM vs. LTMM; ^f^*p* < 0.05, female stage III–IV non–LTMM vs. LTMM; ^g^*p* < 0.05, all subjects non–LTMM vs. LTMM.

Data are presented as median (IQR). Superscripts (a–g) indicate *p* < 0.05 for the specified comparisons; absence of a superscript indicates *p* ≥ 0.05.

T4MI, thoracic 4th vertebra level muscle index; T4CSA, thoracic 4th vertebra level cross–sectional area; LTMM, low thoracic muscle mass; Non–LTMM, non–low thoracic muscle mass.

**Figure 3:**
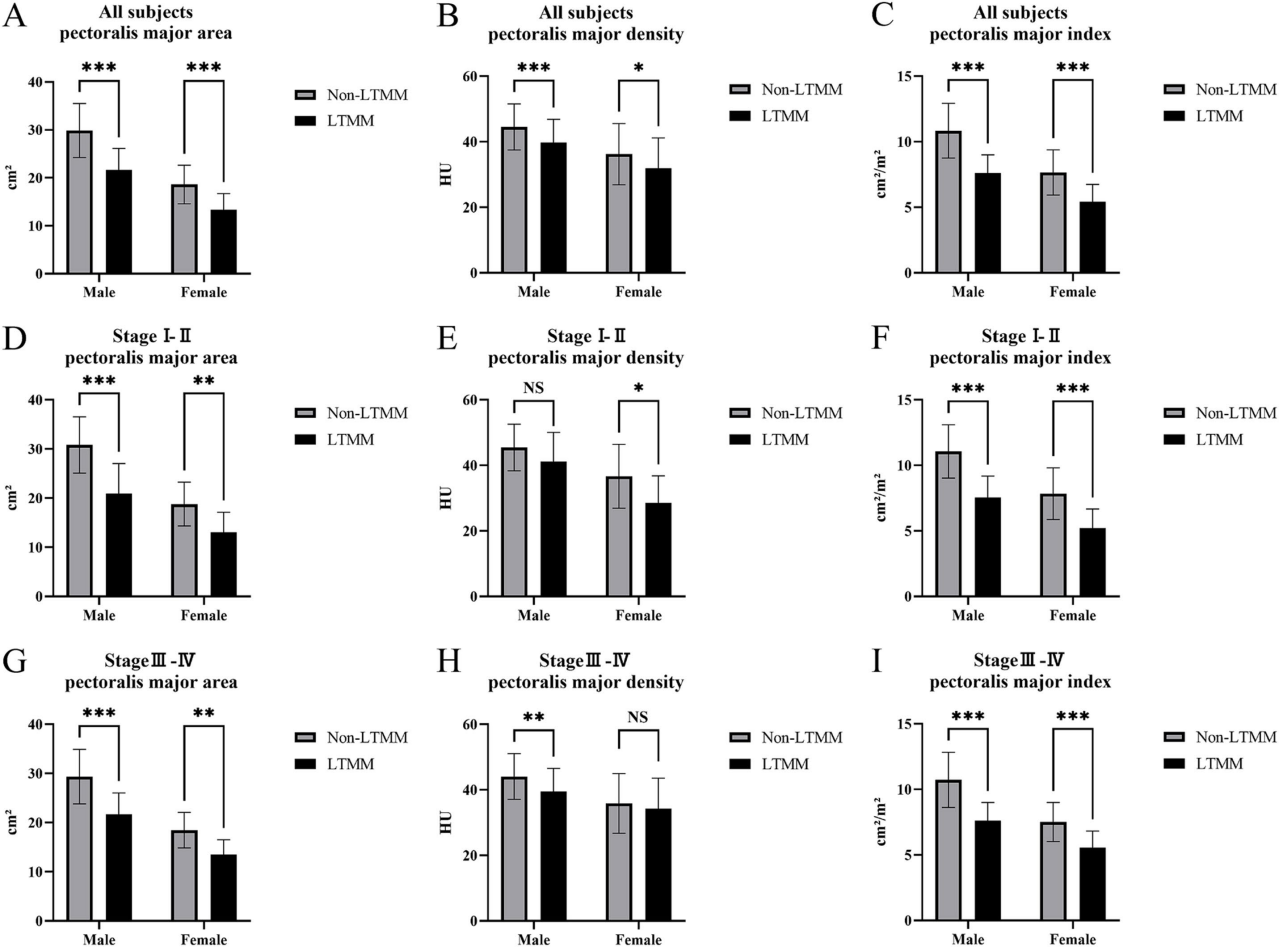
Pectoralis major parameters categorized by sex, clinical stage, and thoracic muscle mass status. Bar plots compare area (left column), density (middle column), and index (area/height^2^; right column) of the pectoralis major between non–LTMM (gray) and LTMM (black). Panels **(A–C)** show all subjects, **(D–F)** stage I–II, and **(G–I)** stage III–IV; within each panel results are shown separately for males and females. Units: area = cm^2^, density = HU, index = cm^2^/m^2^. Significance: *p* < 0.05 (*), *p* < 0.01 (**), *p* < 0.001 (***), NS, not significant; LTMM, low thoracic muscle mass; non–LTMM, non–low thoracic muscle mass.

Male and female patients were stratified separately into early–stage (stage I–II) non–LTMM and LTMM subgroup, as well as advanced–stage (stage III–IV) non–LTMM and LTMM subgroup. Among male participants, the early–stage subgroup comprised 48 patients with non–LTMM and 5 with LTMM, and the advanced–stage subgroup comprised 93 and 41 subjects, respectively. Among female participants, the early–stage subgroup included 35 patients with non–LTMM and 10 with LTMM, and the advanced–stage subgroup included 42 and 15 subjects, respectively. In advanced–stage male and early–stage female participants, all muscle parameters (area, density, and index) were significantly lower in the LTMM group than in the non–LTMM group (all *p* < 0.05). In early–stage male participants, all parameters differed significantly except the densities of the pectoralis major, pectoralis minor, and chest wall muscle group (*p* > 0.05); a similar pattern was observed in advanced–stage female participants. Pectoralis major muscle parameters stratified by gender, clinical stage and LTMM status were provided as examples (Figure 3).

### 3.3 Discriminatory performance of muscle parameters for low thoracic muscle mass in the overall lung cancer cohort

**Figure 4:**
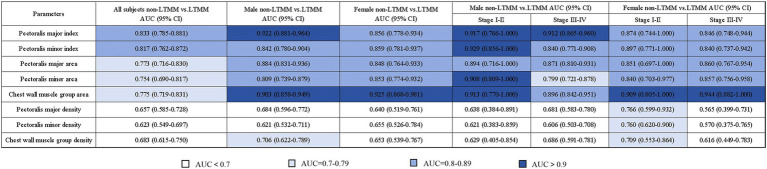
Discriminatory performance (AUC with 95% CI) of T4–level muscle parameters for distinguishing low versus non–low thoracic muscle mass status defined by sex–specific cutoffs. Columns summarize the overall cohort and sex– and stage–specific strata (male/female; stage I–II and stage III–IV). Color intensity reflects AUC magnitude (lighter = lower, darker = higher). AUC interpretation: >0.90 = excellent; 0.80–0.89 = good; 0.70–0.79 = fair; <0.70 = poor. The pectoralis major index shows good discrimination in the overall cohort and excellent discrimination in men. LTMM, low thoracic muscle mass; non–LTMM, non–low thoracic muscle mass.

**Figure 5:**
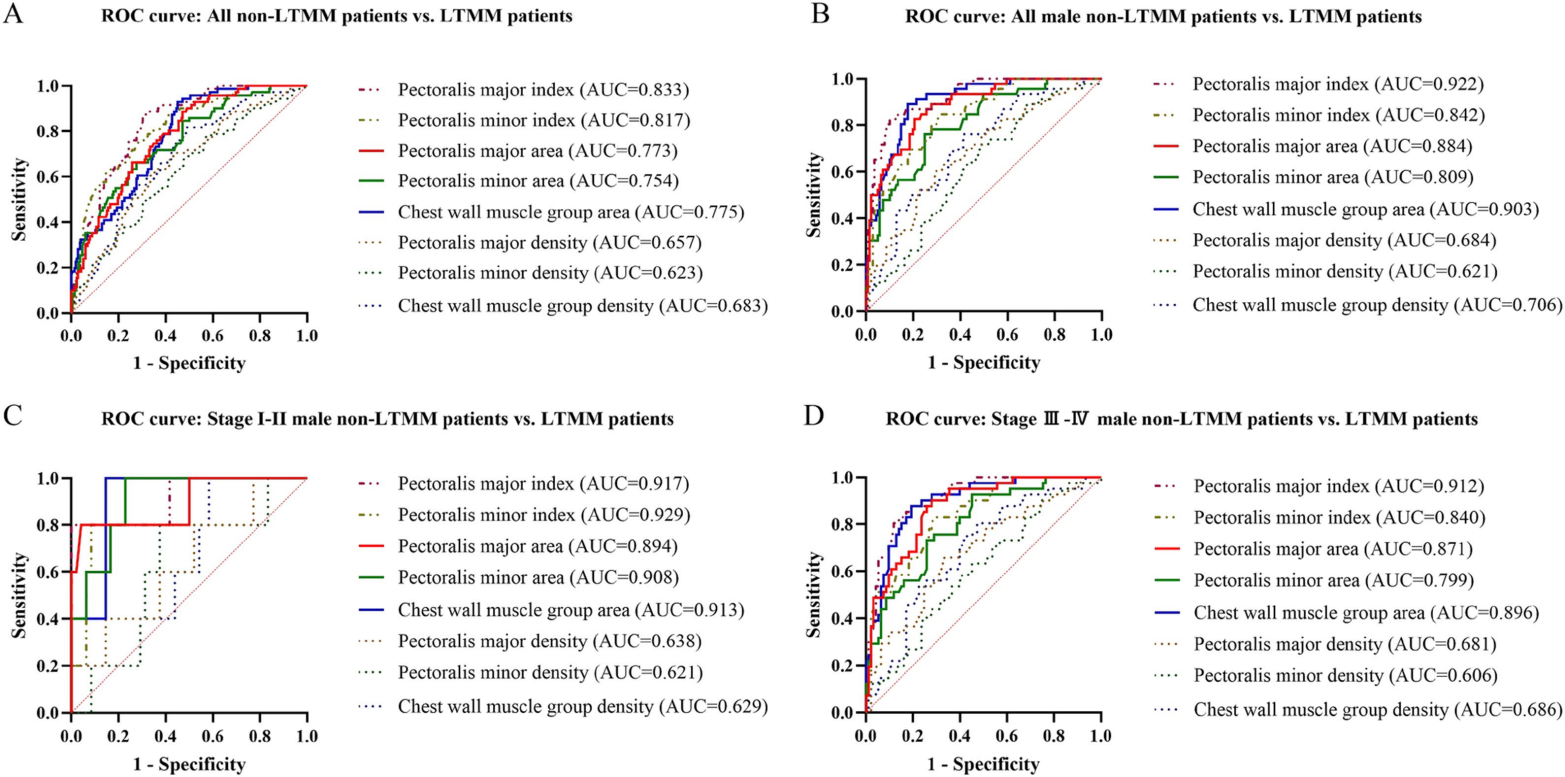
Receiver–operating characteristic (ROC) curves for discriminating LTMM using T4–level muscle parameters in the entire lung cancer patient cohort and the male subgroup. ROC curves compare T4–level muscle parameters for distinguishing non–LTMM vs. LTMM across strata: **(A)** all subjects, **(B)** all men, **(C)** men with stage I–II disease, and **(D)** men with stage III–IV disease. Parameters include pectoralis major/minor index, area, and density, and chest wall muscle area and density. The pectoralis major index achieves the highest overall discriminatory performance (AUC = 0.833) and showed superior discrimination in all men (AUC = 0.922), men with stage I–II disease (AUC = 0.917), and men with stage III–IV disease (AUC = 0.912). LTMM, low thoracic muscle mass; non–LTMM, non–low thoracic muscle mass.

Using sex–specific 25th–percentile T4MI thresholds (40.78 cm^2^/m^2^ for men; 33.15 cm^2^/m^2^ for women), we generated the corresponding AUC values (Figure 4) and representative ROC curves (Figure 5) for each muscle parameter to discriminate low from non–low thoracic muscle mass status. Discriminatory performance was interpreted by area under the ROC curve (AUC) as follows: >0.90, excellent; 0.80–0.89, good; 0.70–0.79, fair; <0.70, poor.

In the overall cohort, the AUCs ranged from 0.623 (95% CI: 0.585–0.728) to 0.833 (95% CI: 0.785–0.881), sensitivities from 61.97 to 92.96%, and specificities from 43.12 to 85.32%. Overall, muscle indices demonstrated excellent discriminatory performance compared with cross–sectional area, which in turn outperformed muscle density. It is noteworthy that the pectoralis major index achieved the highest AUC value [0.833 (95% CI: 0.785–0.881)], demonstrating good discriminatory performance, with sensitivity and specificity of 88.73 and 68.35%, respectively.

Among all male subjects, the AUCs ranged from 0.621 (95% CI: 0.532–0.711) to 0.922 (95% CI: 0.881–0.964), sensitivities from 60.87 to 89.13%, and specificities from 51.06 to 90.07%. Consistent across all patient groups, the pectoralis major index reached the highest AUC value [0.922 (95% CI: 0.881–0.964)], demonstrating superior discriminatory performance, with sensitivity and specificity of 82.61 and 90.07%, respectively. In comparison, among female subjects, the AUCs ranged from 0.640 (95% CI: 0.519–0.761) to 0.925 (95% CI: 0.868–0.981), sensitivities from 56 to 92%, and specificities from 49.35 to 88.31%. The chest wall muscle area achieved the highest AUC value [0.925 (95% CI: 0.868–0.981)], demonstrating excellent discriminatory performance, with the sensitivity and specificity of 88.00 and 88.31%, respectively.

### 3.4 Discriminatory performance of muscle parameters for low thoracic muscle mass across different clinical stages of lung cancer

In early–stage male subgroup, the AUCs ranged from 0.621 (95% CI: 0.383–0.859) to 0.929 (95% CI: 0.856–1.000), sensitivities from 80.00 to 99.99%, and specificities from 41.67 to 99.99%. The pectoralis major index, pectoralis minor index, pectoralis minor area and chest wall muscle area demonstrated superior discriminatory performance, with the AUCs ranging from 0.908 (95% CI: 0.809–1.000) to 0.929 (95% CI: 0.856–1.000), sensitivities ranging from 80.00 to 99.99%, and specificities ranging from 77.08 to 99.99%. In advanced–stage male subgroup, the AUCs ranged from 0.606 (95% CI: 0.503–0.708) to 0.912 (95% CI: 0.865–0.960), sensitivities from 63.41 to 90.24%, and specificities from 52.69 to 86.02%. Compared with other muscle parameters, the pectoralis major index was the only muscle parameter that achieved excellent discriminatory performance, with an AUC value of 0.912 (95% CI: 0.865–0.960) and sensitivity and specificity of 82.93 and 86.02%, respectively.

In early and advanced–stage female subgroup, the AUCs ranged from 0.565 (95% CI: 0.399–0.731) to 0.944 (95% CI: 0.882–1.000), sensitivities from 60.00 to 99.99%, and specificities from 45.71 to 94.00%. The chest wall muscle area demonstrated excellent discriminatory performance in both early [AUC = 0.909 (0.805–1.000); sensitivity = 80%; specificity = 94.29%] and advanced stages [AUC = 0.944 (95% CI: 0.882–1.000); sensitivity = 93.33%; specificity = 85.71%]. By contrast, pectoralis major/minor area and index showed good performance across stages, with the AUCs ranging from 0.840 (95% CI: 0.703–0.977) to 0.897 (95% CI: 0.771–1.000). Notably, several sex- and stage-stratified subgroups were small, resulting in wider confidence intervals; therefore, these stratified AUC estimates should be interpreted as exploratory.

Sex–specific differences could reflect underlying muscle biology and measurement stability. In men, the larger pectoralis major and its greater hormone– and type II fiber–related responsiveness might make the pectoralis major index a more sensitive surrogate of overall muscle reserve. In women, the smaller pectoralis major may be more susceptible to single–muscle variability, whereas the composite chest wall muscle group might offer a more stable marker of thoracic reserves. This could partly explain the observed sex–specific differences in AUC values.

### 3.5 Factors associated with poor performance status defined by ECOG score

**Figure 6:**
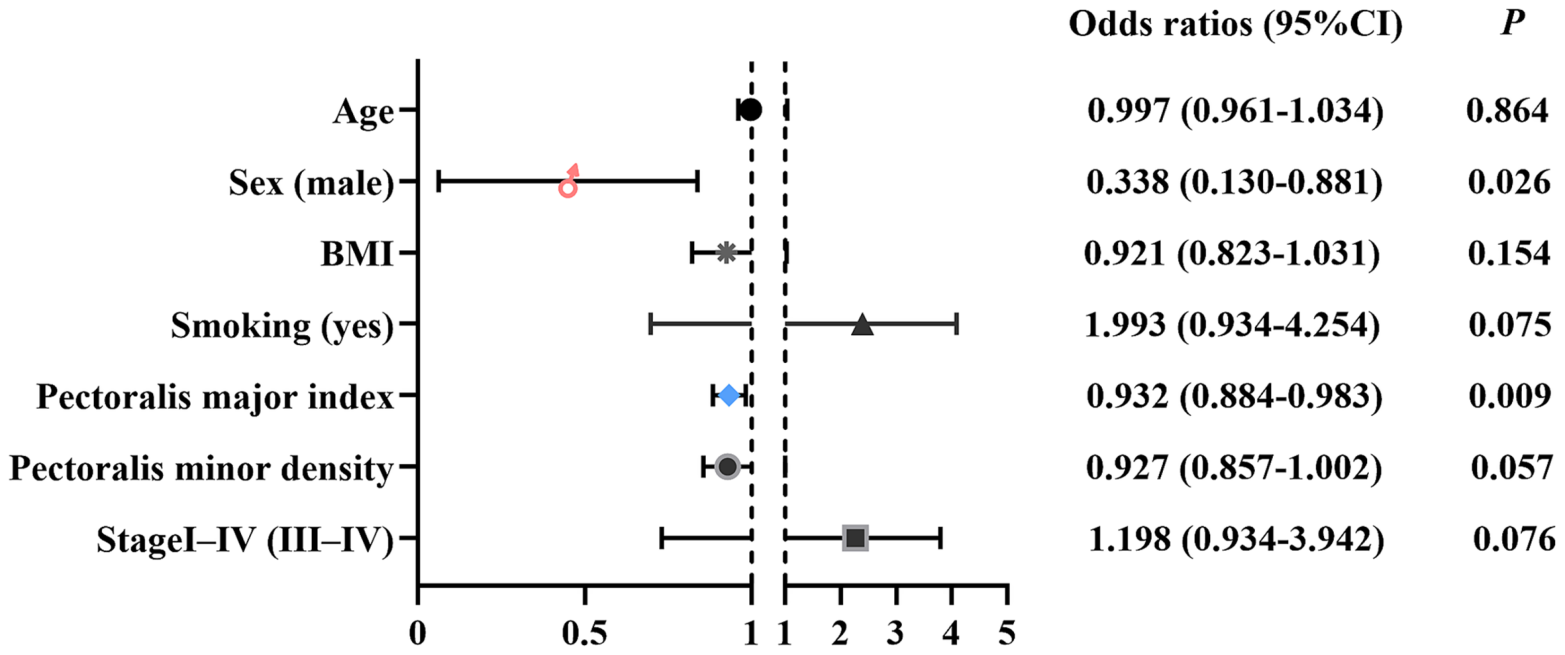
Forest plot of factors independently associated with poor ECOG performance status. Multivariable logistic regression adjusting for age, sex, BMI, smoking status, clinical stage, pectoralis major index, and pectoralis minor density shows adjusted ORs (95% CIs). A lower pectoralis major index was associated with higher odds of poor ECOG status (OR = 0.932; 95% CI 0.884–0.983; *p* = 0.009), and male sex was also significant (OR = 0.338; 95% CI 0.130–0.881; *p* = 0.026). ECOG, Eastern Cooperative Oncology Group; OR, odds ratio; CI, confidence interval.

Table 3 summarizes the univariable associations with poor ECOG performance status (ECOG ≥ 2). Clinically relevant covariates (age, sex, BMI, smoking status, and clinical stage) were included *a priori*, and variables significant in univariable analyses were additionally entered into the multivariable model. Subsequently, in multivariable logistic regression, the pectoralis major index was independently associated with ECOG status (OR = 0.932, 95% CI 0.884–0.983; *p* = 0.009), indicating lower odds of poor ECOG status with a higher index. Male sex was associated with lower odds of poor ECOG status compared with female sex (OR = 0.338, 95% CI 0.130–0.881; *p* = 0.026) (Figure 6). Model calibration was evaluated using the Hosmer–Lemeshow goodness–of–fit test and the Brier score; the Hosmer–Lemeshow test was non–significant (*χ*^2^ = 6.633, df = 8, *p* = 0.577) and the Brier score was 0.131.

**Table 3:** Logistic regression of factors associated with poor performance status (ECOG ≥2).

Variable	Univariable regression		Multivariable regression		
	*p*	Odds ratio (95% CI)	*p*	Odds ratio (95% CI)	VIF
Age (year)	0.178	1.023 (0.990–1.057)	0.864	0.997 (0.961–1.034)	1.270
Sex (male)	0.036	0.475 (0.237–0.951)	0.026	0.338 (0.130–0.881)	1.616
BMI (kg/m^2^)	0.648	0.978 (0.888–1.077)	0.154	0.921 (0.823–1.031)	1.069
Smoking status (yes)	0.009	2.226 (1.218–4.069)	0.075	1.993 (0.934–4.254)	1.411
Pectoralis major index (cm^2^/m^2^)	0.004	0.941 (0.902–0.981)	0.009	0.932 (0.884–0.983)	1.503
Pectoralis minor density (HU)	0.011	0.928 (0.877–0.983)	0.057	0.927 (0.857–1.002)	1.700
Stage I–II vs. stage III–IV (III–IV)	0.741	1.111 (0.596–2.071)	0.076	1.198 (0.934–3.942)	1.038
Height (cm)	0.417	1.017 (0.977–1.059)	–	–	–
Weight (kg)	0.865	1.003 (0.973–1.034)	–	–	–
Pectoralis major area (cm^2^)	0.547	1.012 (0.974–1.052)	–	–	–
Pectoralis minor area (cm^2^)	0.335	1.069 (0.933–1.224)	–	–	–
Chest wall muscle group area (cm^2^)	0.601	1.005 (0.987–1.023)	–	–	–
Pectoralis major density (HU)	0.314	0.984 (0.952–1.016)	–	–	–
Chest wall muscle group density (HU)	0.148	0.961 (0.925–1.010)	–	–	–
Pectoralis minor index (cm^2^/m^2^)	0.517	1.143 (0.763–1.712)	–	–	–
T4MI (cm^2^/m^2^)	0.838	1.004 (0.967–1.042)	–	–	–
T4CSA (cm^2^)	0.535	1.004 (0.992–1.016)	–	–	–

Age, sex, BMI, smoking status, and stage were entered a priori; additional variables significant in univariable analyses were also considered.

ECOG, eastern cooperative oncology group performance status; BMI, body mass index; T4MI, thoracic 4th vertebra level muscle index; T4CSA, thoracic 4th vertebra level cross-sectional area; VIF, variance inflation factor.

## 4 Discussion

This retrospective study analyzed T4–level muscle parameters in patients with lung cancer to evaluate their TNM stage–stratified discriminatory performance for low thoracic muscle mass (LTMM) and examine the associations with performance status defined by ECOG score. Our primary findings suggest that measurements of the pectoralis major, pectoralis minor, and chest wall musculature showed good discriminatory performance for lung cancer–related low thoracic muscle mass. We used a sex–specific 25th–percentile cutoff as a pragmatic, data–driven and exploratory operational definition to enable opportunistic risk stratification on chest CT when no universally accepted T4 reference threshold is available. Overall, the pectoralis major index was independently associated with ECOG–defined performance status, with a lower index corresponding to higher odds of poor ECOG status.

As anticipated, muscle cross–sectional area, density, and derived indices were significantly lower in the LTMM group compared to the non–LTMM group. This is consistent with the results of other disease–related sarcopenia, such as COPD, COVID–19, colorectal cancer, gastric cancer and pancreatic cancer (9, 10, 22–24). These alterations in muscle parameters may reflect concurrent deterioration in muscle quantity and quality during cancer–associated sarcopenia. First, chronic inflammation causes myofiber injury and increases intra– and intermuscular fat infiltration, thereby lowering muscle density. In line with this mechanism, muscle density was lower in the LTMM group than in the normal group (e.g., pectoralis major density: 37.13 vs. 42.66 HU; chest wall muscle density: 39.28 vs. 43.23 HU). Second, fatigue, anorexia, and hospitalization reduce mechanical loading and physical activity, leading to disuse atrophy and smaller cross–sectional area. Consistently, cross–sectional areas were smaller in LTMM than in normal muscle mass (e.g., pectoralis major area: 18.56 vs. 26.03 cm^2^; T4CSA: 98.09 vs. 121.14 cm^2^). Third, after adjustment for body size, skeletal muscle indices indicate depletion of intrinsic muscle reserves.

Overall, skeletal muscle indices outperformed cross–sectional muscle area and muscle density in distinguishing low from non–low thoracic muscle mass status. This hierarchy was reflected in our ROC analyses, where index–based metrics generally yielded higher AUCs than area and density measures in the overall cohort and most strata. This agrees with the findings on sarcopenia associated with conditions such as COVID–19 and acute ischemic stroke (9, 25). This pattern may be explained by several underlying factors. Firstly, cross–sectional area reflects absolute muscle quantity but does not account for body size, consequently, it may overestimate muscular reserves in taller or larger individuals. Secondly, muscle density primarily reflects intramuscular fat infiltration and thus serves as an imaging biomarker of muscle quality. However, when considered in isolation it is strongly influenced by CT acquisition parameters, metabolic comorbidities, and hydration status, compromising its stability and discriminatory performance (26). By contrast, the skeletal muscle index normalizes area to height^2^, substantially attenuating body–size confounding, better representing intrinsic muscular reserves and functional status, and thereby improving discriminatory performance. Nonetheless, index–based measures are unavoidably subject to minor residual confounding from systemic inflammation, fluid status, and physical activity (2, 27).

In our study, T4 muscle parameters demonstrated good discriminatory performance for low muscle mass, consistent with reports in colorectal cancer, and diabetes (13, 14, 28). Collectively, these findings reinforce a strong correlation between T4–level musculature and whole–body muscle status, demonstrating the potential for T4 measurements to directly assess low thoracic muscle mass in thoracic imaging. More importantly, as chest CT is not only routinely used for the diagnosis, staging, and follow–up of lung cancer, but also enables quantitative assessment of systemic muscle status without additional radiation exposure or cost. This facilitates the “one–stop–shop” evaluation of tumor burden and patient performance status within a single thoracic examination procedure.

Interestingly, we found that the pectoralis major measurements showed good internal discrimination for low thoracic muscle mass in the lung cancer population, with the pectoralis major index yielding the highest overall AUC (0.833). This aligns with prior chest CT body–composition studies in which pectoral muscle–based indices have demonstrated strong discriminatory or risk–stratification utility, including work by Sun et al. (11, 29–31) and Moon et al. (12) that used pectoral muscle metrics as imaging surrogates of reduced muscle mass or related vulnerability. Physiologically, the pectoralis major, the largest upper limb prime mover on the anterior chest wall and an accessory inspiratory muscle, may serves as a marker of both upper–extremity strength and ventilatory reserve; declines in its mass and density may indicate impairments in these domains. In lung cancer, limitations in exertional capacity and ventilation reduce day–to–day upper limb activity, placing the pectoralis major under chronically low mechanical load and predisposing it to atrophy and fatty infiltration. Besides, fiber–type biology may further explain its distinctive value. It has been demonstrated that Type II fibers are more fatigue–prone than type I fibers (32). Relative to other thoracic muscles, the pectoralis major contains a higher proportion of type II fibers and is more dependent on protein synthesis and androgen signaling, during systemic catabolism or blunted anabolism, it may therefore be more susceptible to atrophy (33, 34). Notably, the pectoralis major index was the only parameter to achieve superior discrimination across both early and advanced stages in male participants (AUC range, 0.912–0.917), whereas in women the chest wall muscle group area showed the highest discrimination (AUC range, 0.909–0.944). In contrast, the chest wall muscle group aggregates multiple respiratory and postural muscles and may therefore provide a more robust composite marker of thoracic muscle reserves in women. This sex difference may reflects higher testosterone levels in men, which favor type II fiber hypertrophy and confer greater baseline upper limb lean mass and strength (34–36). By contrast, women typically exhibit lower cross–sectional area and functional metrics related to type II fibers (35, 37), as well as lower absolute upper limb muscle mass and a smaller proportion of upper limb muscle relative to total body muscle, resulting in comparatively lower upper limb strength (34, 36). Consequently, we speculate that pectoralis major–derived metrics may better reflect whole–body muscle reserves in men, whereas in women the chest wall muscle group, by integrating multiple respiratory and postural muscles, may provide a more stable composite representation of thoracic muscle quantity than the smaller and more variable pectoralis major alone.

Encouragingly, we observed that the pectoralis major index was independently associated with ECOG performance status, which is consistent with our speculation in “Introduction” that pectoralis major–derived measures may reflect functional status in patients with lung cancer. Structurally, as the largest upper limb prime mover on the anterior chest wall and a contributor to inspiration, a diminished pectoralis major may coincide with reduced upper limb work capacity and ventilatory reserve. These aspects are captured in the ECOG scale’s assessment of daily and physical activities. Because pectoralis major cross–sectional area (CSA) is confounded by body size and can therefore be less stable, normalizing CSA to height^2^ (the pectoralis major index) substantially mitigates this bias, better reflects intrinsic muscular reserves and functional status (38), and was independently associated with ECOG–defined performance status in lung cancer. Subsequently, it compensates for CSA’s limitations in capturing functional capacity. Accordingly, the pectoralis major index may serve as a practical indicator of functional status in patients with lung cancer and provide richer information for clinical decision–making.

There are several limitations to this study. First, this retrospective, single-center study included patients from a single ethnic background, which may limit generalizability to some extent. Second, scans were acquired on different CT scanner models, and although we used a harmonized protocol, minor scanner–related variability is difficult to fully avoid. Third, because both the T4MI–based reference and the candidate surrogates were derived from the same T4 level, the discriminatory performance observed in ROC analysis may be overestimated. In addition, some sex- and stage-stratified subgroups were small, which may limit the robustness of subgroup analyses. Moreover, abdominal CT at the L3 level and other independent reference standards were not available in this cohort, precluding validation against an independent reference. Fourth, the cross–sectional design without survival follow–up precluded causal inference and validation of our cutoffs using overall survival and progression-free survival. Lastly, given the small number of patients with ECOG 3 and resulting instability in higher categories, we dichotomized ECOG (0–1 vs. ≥2) and applied binary logistic regression rather than multivariable ordinal regression. Therefore, the analyses should be interpreted as associations with dichotomized ECOG–defined clinical status rather than evidence of an ordinal relationship across the full ECOG scale.

## 5 Conclusion

T4–level musculature demonstrates effective discriminatory capabilities for lung cancer–related low thoracic muscle mass across clinical stages and by sex. Notably, among all muscle parameters analyzed, the pectoralis major index achieved the highest overall discriminatory performance. Furthermore, the pectoralis major index is closely associated with ECOG performance status and may be a potential imaging biomarker associated with functional status in patients with lung cancer.

## Data Availability

The datasets presented in this article are not readily available due to national policies and hospital confidentiality regulations. Requests to access these datasets should be directed to pf520llm@163.com.

## References

[1] Bray F Laversanne M Sung H Ferlay J Siegel RL Soerjomataram I . Global cancer statistics 2022: GLOBOCAN estimates of incidence and mortality worldwide for 36 cancers in 185 countries. CA Cancer J Clin. (2024) 74:229–63. doi: 10.3322/caac.21834, 38572751

[2] Bilski J Szlachcic A Ptak-Belowska A Brzozowski T. Physical activity, exerkines, and their role in cancer cachexia. Int J Mol Sci. (2025) 26:8011. doi: 10.3390/ijms26168011, 40869331 PMC12386252

[3] Koh K Scott R Feliciano EMC Janowitz T Goncalves MD White EP . Cancer-associated cachexia: bridging clinical findings with mechanistic insights in human studies. Cancer Discov. (2025) 15:1543–68. doi: 10.1158/2159-8290.CD-25-0293 40298389 PMC12794921

[4] Jensen S Bloch Z Quist M Hansen TTD Johansen C Pappot H . Sarcopenia and loss of muscle mass in patients with lung cancer undergoing chemotherapy treatment: a systematic review and meta-analysis. Acta Oncol. (2023) 62:318–28. doi: 10.1080/0284186X.2023.2180660 37051865

[5] Prado CM Lieffers JR McCargar LJ Reiman T Sawyer MB Martin L . Prevalence and clinical implications of sarcopenic obesity in patients with solid tumours of the respiratory and gastrointestinal tracts: a population-based study. Lancet Oncol. (2008) 9:629–35. doi: 10.1016/S1470-2045(08)70153-0, 18539529

[6] Hong JH Hong H Choi YR Kim DH Kim JY Yoon JH . CT analysis of thoracolumbar body composition for estimating whole-body composition. Insights Imaging. (2023) 14:69. doi: 10.1186/s13244-023-01402-z, 37093330 PMC10126176

[7] Moon SW Choi JS Lee SH Jung KS Jung JY Kang YA . Thoracic skeletal muscle quantification: low muscle mass is related with worse prognosis in idiopathic pulmonary fibrosis patients. Respir Res. (2019) 20:35. doi: 10.1186/s12931-019-1001-6 30767787 PMC6376641

[8] Jennerich AL Downey L Goss CH Kapnadak SG Pryor JB Ramos KJ. Computed tomography body composition and clinical outcomes following lung transplantation in cystic fibrosis. BMC Pulm Med. (2023) 23:105. doi: 10.1186/s12890-023-02398-4, 36997883 PMC10062009

[9] Wen Z Wang T Luo S Liu Y. CT scan-derived pectoralis muscle parameters are closely associated with COVID-19 outcomes: a systematic review and meta-analysis. PLoS One. (2025) 20:e0316893. doi: 10.1371/journal.pone.0316893, 39874384 PMC11774355

[10] McDonald MLN Diaz AA Rutten E Lutz SM Harmouche R San Jose Estepar R . Chest computed tomography-derived low fat-free mass index and mortality in COPD. Eur Respir J. (2017) 50:1701134. doi: 10.1183/13993003.01134-2017, 29242259 PMC5890424

[11] Sun C Anraku M Kawahara T Karasaki T Kitano K Nagayama K . Prognostic significance of low pectoralis muscle mass on preoperative chest computed tomography in localized non-small cell lung cancer after curative-intent surgery. Lung Cancer. (2020) 147:71–6. doi: 10.1016/j.lungcan.2020.07.008, 32673829

[12] Moon SW Lee SH Woo A Leem AY Lee SH Chung KS . Reference values of skeletal muscle area for diagnosis of sarcopenia using chest computed tomography in Asian general population. J Cachexia Sarcopenia Muscle. (2022) 13:955–65. doi: 10.1002/jcsm.12946, 35170229 PMC8978009

[13] Tao J Shi H Shen B Zhang L Tu Y Zhang X. The chest CT perspective on sarcopenia: exploring reference values for muscle mass quantity/quality and its application in elderly adults. Nutrition. (2024) 128:112558. doi: 10.1016/j.nut.2024.112558, 39276682

[14] Arayne AA Gartrell R Qiao J Baird PN Yeung JM. Comparison of CT derived body composition at the thoracic T4 and T12 with lumbar L3 vertebral levels and their utility in patients with rectal cancer. BMC Cancer. (2023) 23:56. doi: 10.1186/s12885-023-10522-0, 36647027 PMC9843961

[15] O’Brien ME Zou RH Hyre N Leader JK Fuhrman CR Sciurba FC . CT pectoralis muscle area is associated with DXA lean mass and correlates with emphysema progression in a tobacco-exposed cohort. Thorax. (2023) 78:394–401. doi: 10.1136/thoraxjnl-2021-217710 34853157 PMC9156725

[16] Wu FZ Huang YL Wu YJ Tang EK Wu MT Chen CS . Prognostic effect of implementation of the mass low-dose computed tomography lung cancer screening program: a hospital-based cohort study. Eur J Cancer Prev. (2020) 29:445–51. doi: 10.1097/CEJ.0000000000000569, 32740170

[17] Amin MB Greene FL Edge SB Compton CC Gershenwald JE Brookland RK . The eighth edition AJCC cancer staging manual: continuing to build a bridge from a population-based to a more “personalized” approach to cancer staging. CA Cancer J Clin. (2017) 67:93–9. doi: 10.3322/caac.21388, 28094848

[18] Jogiat UM Bédard A Baracos V Turner SR Eurich D Filafilo H . Thoracic muscle mass predicts survival among patients with locally advanced esophageal cancer. Clin Nutr. (2025) 49:90–7. doi: 10.1016/j.clnu.2025.03.017, 40253811

[19] McDonald F De Waele M Hendriks LEL Faivre-Finn C Dingemans AMC Van Schil PE. Management of stage I and II nonsmall cell lung cancer. Eur Respir J. (2017) 49:1600764. doi: 10.1183/13993003.00764-2016, 28049169

[20] Mischel AM Rosielle DA. Eastern cooperative oncology group performance status #434. J Palliat Med. (2022) 25:508–10. doi: 10.1089/jpm.2021.0599 35230903

[21] Chalker C Voutsinas JM Wu QV Santana-Davila R Hwang V Baik CS . Performance status (PS) as a predictor of poor response to immune checkpoint inhibitors (ICI) in recurrent/metastatic head and neck cancer (RMHNSCC) patients. Cancer Med. (2022) 11:4104–11. doi: 10.1002/cam4.4722, 35349227 PMC9678089

[22] He X Zhou S Li H Gou Y Jia D. Prognostic role of pretreatment skeletal muscle index in gastric cancer patients: a meta-analysis. Pathol Oncol Res. (2023) 29:1611055. doi: 10.3389/pore.2023.1611055, 37168049 PMC10164928

[23] Lee MH Pickhardt SG Garrett JW Perez AA Zea R Valle KF . Utility of fully automated body composition measures on pretreatment abdominal CT for predicting survival in patients with colorectal cancer. AJR Am J Roentgenol. (2023) 220:371–80. doi: 10.2214/AJR.22.28043, 36000663

[24] Raoul P Cintoni M Coppola A Alfieri S Tortora G Gasbarrini A . Preoperative low skeletal muscle mass index assessed using L3-CT as a prognostic marker of clinical outcomes in pancreatic cancer patients undergoing surgery: a systematic review and meta-analysis. Int J Surg. (2024) 110:6126–34. doi: 10.1097/JS9.0000000000000989, 38836800 PMC11486987

[25] Yilmaz E Sarier IF Gocmen R Arsava EM Topcuoglu MA. Pectoralis major muscle index as an opportunistic predictor of mortality in acute stroke patients treated with intravenous thrombolysis. Neurol Sci. (2025) 46:2195–202. doi: 10.1007/s10072-025-08026-9, 39976881 PMC12003620

[26] Lin YH Tsai PS Hung CL Beg MF Yeh HI Yun CH . The role of muscle density in predicting the amputation risk in peripheral arterial disease: a tissue composition study using lower extremity CT angiography. Diagnostics (Basel). (2025) 15:1439. doi: 10.3390/diagnostics15111439, 40507011 PMC12154033

[27] Golder AM Ferguson M McMillan P Mansouri D Horgan PG Roxburgh CS . CT-derived body composition and differential association with age, TNM stage and systemic inflammation in patients with colon cancer. Sci Rep. (2024) 14:15673. doi: 10.1038/s41598-024-65871-y, 38977870 PMC11231341

[28] Zhang L Shi H Shen B Tao J Tu Y Zhang X. Diabetes is associated with impaired skeletal muscle quality, not quantity, in older adults, based on CT-derived chest muscle metrics. Maturitas. (2025) 202:108710. doi: 10.1016/j.maturitas.2025.108710, 40907340

[29] Sun C Hirata Y Kawahara T Kawashima M Sato M Nakajima J . Diagnosis of respiratory sarcopenia for stratifying postoperative risk in non–small cell lung cancer. JAMA Surg. (2025) 160:66–73. doi: 10.1001/jamasurg.2024.4800, 39475952 PMC11581747

[30] Sun C Anraku M Kawahara T Kawashima M Sato M Nakajima J . Respiratory strength and pectoralis muscle mass as measures of sarcopenia: relation to outcomes in resected non–small cell lung cancer. J Thorac Cardiovasc Surg. (2022) 163:779–787.e2. doi: 10.1016/j.jtcvs.2020.10.133 33317785

[31] Sun C Anraku M Kawahara T Karasaki T Konoeda C Kitano K . Combination of skeletal muscle mass and density predicts postoperative complications and survival of patients with non-small cell lung cancer. Ann Surg Oncol. (2022) 29:1816–24. doi: 10.1245/s10434-021-11024-8, 34997412

[32] Schiaffino S Reggiani C. Fiber types in mammalian skeletal muscles. Physiol Rev. (2011) 91:1447–531. doi: 10.1152/physrev.00031.2010, 22013216

[33] Bhasin S. Mechanisms of testosterone’s anabolic effects on muscle and function: controversies and new insights. Endocr Rev. (2025):bnaf041. doi: 10.1210/endrev/bnaf041 41355050 PMC13017102

[34] Nuzzo JL. Narrative review of sex differences in muscle strength, endurance, activation, size, fiber type, and strength training participation rates, preferences, motivations, injuries, and neuromuscular adaptations. J Strength Cond Res. (2023) 37:494–536. doi: 10.1519/JSC.0000000000004329, 36696264

[35] Nuzzo JL. Sex differences in skeletal muscle fiber types: a meta-analysis. Clin Anat. (2024) 37:81–91. doi: 10.1002/ca.24091, 37424380

[36] Hunter SK Senefeld JW. Sex differences in human performance. J Physiol. (2024) 602:4129–56. doi: 10.1113/JP284198, 39106346

[37] Haizlip KM Harrison BC Leinwand LA. Sex-based differences in skeletal muscle kinetics and fiber-type composition. Physiology (Bethesda). (2015) 30:30–9. doi: 10.1152/physiol.00024.2014, 25559153 PMC4285578

[38] Derstine BA Holcombe SA Ross BE Wang NC Su GL Wang SC. Optimal body size adjustment of L3 CT skeletal muscle area for sarcopenia assessment. Sci Rep. (2021) 11:279. doi: 10.1038/s41598-020-79471-z, 33431971 PMC7801425

